# Structural Analysis of Mitochondrial Dynamics—From Cardiomyocytes to Osteoblasts: A Critical Review

**DOI:** 10.3390/ijms23094571

**Published:** 2022-04-20

**Authors:** Daniel H. Mendelsohn, Katja Schnabel, Andreas Mamilos, Samuel Sossalla, Steffen Pabel, Georg Daniel Duerr, Karsten Keller, Volker H. Schmitt, Friedrich Barsch, Nike Walter, Ronald Man Yeung Wong, Thaqif El Khassawna, Tanja Niedermair, Volker Alt, Markus Rupp, Christoph Brochhausen

**Affiliations:** 1 Institute of Pathology, University Regensburg, 93053 Regensburg, Germany; daniel.mendelsohn@stud.uni-regensburg.de (D.H.M.); katja.schnabel@stud.uni-regensburg.de (K.S.); andreas.mamilos@ukr.de (A.M.); tanja.niedermair@ukr.de (T.N.); 2 Central Biobank Regensburg, University Regensburg, University Hospital Regensburg, 93053 Regensburg, Germany; 3 Department of Trauma Surgery, University Medical Centre Regensburg, 93053 Regensburg, Germany; nike.walter@ukr.de (N.W.); volker.alt@ukr.de (V.A.); markus.rupp@ukr.de (M.R.); 4 Department of Internal Medicine II, University Hospital Regensburg, 93053 Regensburg, Germany; samuel.sossalla@ukr.de (S.S.); steffen.pabel@ukr.de (S.P.); 5 Department of Cardiovascular Surgery, University Medical Center Mainz (Johannes Gutenberg-University Mainz), 55131 Mainz, Germany; danduerr@uni-mainz.de; 6 Department of Cardiology, Cardiology I, University Medical Center Mainz (Johannes Gutenberg-University Mainz), 55131 Mainz, Germany; karsten.keller@unimedizin-mainz.de (K.K.); volker.schmitt@unimedizin-mainz.de (V.H.S.); 7 Center for Thrombosis and Hemostasis (CTH), University Medical Center Mainz (Johannes Gutenberg-University Mainz), 55131 Mainz, Germany; 8 Department of Sports Medicine, Medical Clinic VII, University Hospital Heidelberg, 69120 Heidelberg, Germany; 9 German Center for Cardiovascular Research (DZHK), Partner Site Rhine Main, 55131 Mainz, Germany; 10 Institute for Exercise and Occupational Medicine, Faculty of Medicine, Medical Center, University of Freiburg, 79106 Freiburg, Germany; friedrich.barsch@uniklinik-freiburg.de; 11 Department of Orthopaedics and Traumatology, Prince of Wales Hospital, The Chinese University of Hong Kong, Hong Kong, China; ronaldwong@ort.cuhk.edu.hk; 12 Department of Experimental Trauma Surgery, Justus-Liebig-University Giessen, 35390 Giessen, Germany; thaqif.elkhassawna@chiru.med.uni-giessen.de

**Keywords:** mitochondria, fission, fusion, ultrastructure, heart, bone, hypoxia, inflammation

## Abstract

Mitochondria play a crucial role in cell physiology and pathophysiology. In this context, mitochondrial dynamics and, subsequently, mitochondrial ultrastructure have increasingly become hot topics in modern research, with a focus on mitochondrial fission and fusion. Thus, the dynamics of mitochondria in several diseases have been intensively investigated, especially with a view to developing new promising treatment options. However, the majority of recent studies are performed in highly energy-dependent tissues, such as cardiac, hepatic, and neuronal tissues. In contrast, publications on mitochondrial dynamics from the orthopedic or trauma fields are quite rare, even if there are common cellular mechanisms in cardiovascular and bone tissue, especially regarding bone infection. The present report summarizes the spectrum of mitochondrial alterations in the cardiovascular system and compares it to the state of knowledge in the musculoskeletal system. The present paper summarizes recent knowledge regarding mitochondrial dynamics and gives a short, but not exhaustive, overview of its regulation via fission and fusion. Furthermore, the article highlights hypoxia and its accompanying increased mitochondrial fission as a possible link between cardiac ischemia and inflammatory diseases of the bone, such as osteomyelitis. This opens new innovative perspectives not only for the understanding of cellular pathomechanisms in osteomyelitis but also for potential new treatment options.

## 1. Introduction

Recent research focuses on the key roles of mitochondria in different pathomechanisms. Existing literature underlines mitochondrial ultrastructure and pathologies as a hot topic in this context [1,2]. Mitochondria are not only crucial for energy production in cells but are also involved in several physiologic processes, such as regulation of apoptosis, calcium handling, innate immunity, and phospholipid synthesis. Energy production is one of the most important functions of the mitochondria. Not surprisingly, clinical evidence shows that energy-intensive cells such as cardiomyocytes are particularly vulnerable to mitochondrial dysfunction [3].

The dysfunction of mitochondria involves changes in the dynamics of mitochondria, such as the fission and fusion processes. The fusion process results in the formation of one mitochondrion out of two, whereas fission describes the division of one mitochondrion into two separate ones. Notably, fission is also a prerequisite for mitophagy [4]. Fission is mostly dependent on the dynamin related protein 1 (Drp1), which is the main component of the fission machinery [5]. Fusion mainly depends on the Mitofusin 1 and 2 (Mfn1 and Mfn2) as well as the GTPase Optic atrophy 1 (Opa1) [6,7]. Both processes are highly linked to each other and can be regulated by multiple factors, such as cellular stress factors, namely, mitochondrial reactive oxygen species (mROS) and mitochondrial DNA damage [3]. Recently, great efforts are performed in research on mitochondria dynamics, mainly focusing on neurons and cardiomyocytes. A PubMed request revealed 318 papers available on mitochondrial fission and fusion in the cardiovascular system since 2017. In contrast, in the same time frame, only 31 papers describe the same process in the musculoskeletal system, namely, in osteoblasts. In fact, mitochondria in other tissues or cell types than in the cardiovascular system have been much less investigated. In consequence, the specific role of mitochondria in several homeostatic and pathophysiological processes is not well understood in these cells and tissues. However, growing evidence arises regarding their active role not only in the immune response but also in the response to pharmaceutic active agents [8,9,10]. Especially within the cardiovascular system, some experimental pharmacological active agents already exist that influence fission and fusion with relevant effects on cells and tissue [11,12].

In the present review, we summarize findings on mitochondrial dynamic processes from the cardiovascular system and question if they could be transformed into the pathophysiology of osteoblasts. Our aim is to better understand the underlying pathomechanisms in this cell type and to shed light on potential new therapeutic strategies for different pathologies of the bone.

## 2. Mitochondrial Ultrastructure and the Repertoire of Ultrastructural Changes

Structurally, mitochondria consist of an outer and an inner membrane. The inner membrane has multiple involutions, forming the so-called cristae. The space between the outer and inner membrane is called the intermembranous space. The inner membrane encloses the mitochondrial matrix, displaying a higher density than the intermembranous space. Each compartment, including the intermembranous space and the matrix, has its own functions and its own set of enzymes responsible for these functions. In this context, the inner membrane contains the enzymes of oxidative phosphorylation, whereas enzymes of the Krebs cycle and fatty acid oxidation are found in the matrix [13,14,15]. Furthermore, certain electron-dense granules can occasionally be found in the matrix. These are thought to be solid-phase calcium stores [16].

Diverse functional changes and lesions result in a structural correlate within the mitochondrial morphology. Thus, there is a repertoire of structural changes in the different mitochondrial compartments. One of the most important alterations is given by different types, orientations, sizes, and concentrations of cristae. The concentration of cristae within mitochondria is linked to the metabolic activity of the corresponding tissue [17]. As an example, continuously active muscle cells from the heart or the diaphragm include plenty of large mitochondria densely packed with multiple cristae [18,19]. Paradoxically, cancer cells display vastly lower numbers of mitochondrial cristae despite their fast growth. This phenomenon can be explained by the typical shift towards anaerobic glycolysis in the cytoplasm of cancer cells, which does not involve mitochondria [20,21].

The most frequent structural type of cristae in mammals is the lamellar type. These lamellar cristae arise from the inner membrane as shelf-like or plate-like infoldings and reach variable lengths. In hepatocytes, cristae barely reach the center of the mitochondrion, whereas cristae in renal or muscle cells extend to almost the entire width of the organelle. Furthermore, in mammals, tubular cristae are found in steroid-producing tissues [22].

**Figure 1:**
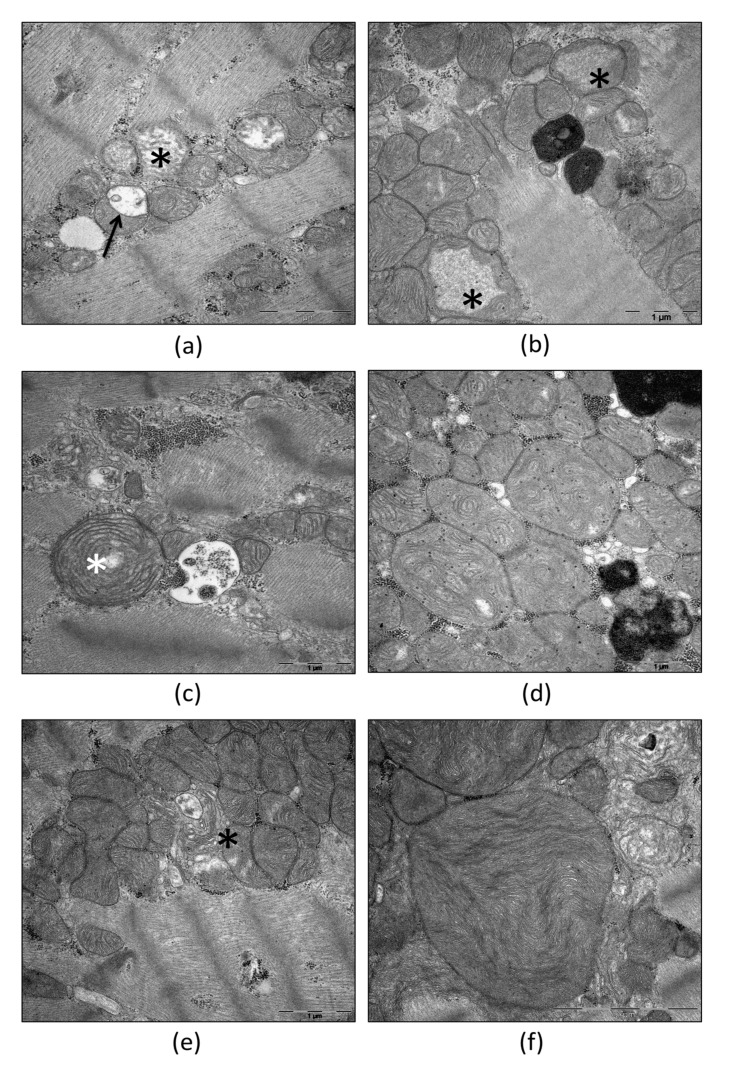
Transmission electron microscopic images of morphological alterations in mitochondria in human skeletal muscle with mitochondriopathy (**a**) and human hypertrophic heart (**b**–**f**). (**a**) Partition (arrow) and swollen hypodense mitochondria (star). (**b**) Swollen mitochondria including cristae dissolution (stars). (**c**) Concentric cristae (star). (**d**) Mitochondria including undulated cristae. (**e**) Cytoplasmic tubules involved in de novo biogenesis (star) (**f**) Megamitochondrium. Institute of Pathology, Regensburg.

Following the increased metabolic activity, for example, due to inflammation, more and more morphological correlates accumulate as signs of mitochondrial damage and degradation. These are increased fission, vacuolar degeneration, or de novo biosynthesis of mitochondria, respectively [23,24]. Furthermore, changes in cristae structure can occur (Figure 1d), such as undulating cristae [25]. The typical correlate of vacuolar degeneration of mitochondria is the formation of concentric cristae (Figure 1c) in the early stages, followed by dissolution of cristae, mitochondrial swelling (Figure 1a,b), and finally the degeneration of autophagosomes. The latter process is called mitophagy [23,26].

It is well known that mitochondria are, in many cases, associated with the tubules of the rough endoplasmic reticulum (RER). The contact sites between mitochondria and the RER are responsible for the communication between these two organelles and play a relevant functional role [27,28]. In this context, the transfer of calcium from the RER to mitochondria takes place at these contact sites. Increased tethering between the two organelles leads to mitochondrial swelling and cell death [29]. Furthermore, these contact sites are the proposed localization of mitochondrial fission [30]. While fission describes the formation of new mitochondria from existing ones, de novo biosynthesis describes the formation of new mitochondria from the cytoplasm. The morphological correlate of the de novo biosynthesis (Figure 1e) is the invagination of cytoplasmic processes containing vesicles and tubules into large cytoplasmic vacuoles [31].

A morphological approach to analyzing fission and fusion in different cell types is given by ultrastructural analyses by the use of transmission electron microscopy. In this context, fission has the following two different ultrastructural correlates: partitions and pinching. Partitions (Figure 1a) have frequently been described for mitochondria in various mammalian cell types. They are comprised of the extension of a single crista until it connects with the inner membrane on the opposing side. Subsequently, invagination of the outer membrane ultimately results in the division of the mitochondrion [32]. Interestingly, the subcompartments on both sides of the partition display slightly differently orientated cristae and an altered matrix density. Furthermore, mitochondria appear moderately swollen during partitioning [33]. The fact that this process is related to fission was elucidated by the work of Tandler and coworkers already in the late sixties of the last century [34]. In their experiments, hepatic mitochondria achieved giant sizes following a riboflavin-free diet. This was reversed by the addition of the missing vitamin. Since mitochondria have to divide in order to achieve smaller sizes, it is evident that the abundant partitions displayed in the recovery phase are associated with mitochondrial fission. In an electron microscope study of rat cardiomyocytes, Fujioka and colleagues found one partitioning mitochondrion in >2000 mitochondria under physiological conditions, which was considered an underestimate since partitioned mitochondria could only be identified if they were sectioned at the correct angle [33].

The second morphological correlate of mitochondrial fission is constituted by pinching [32]. This process involves the constriction of mitochondria at certain sites. These constrictions occur at contact sites between the sarcoplasmic reticulum (SR) and the mitochondria. Despite these processes happening simultaneously in vivo, only one of them can be induced at a time following specific stimuli in vitro. In this context, recovery of megamitochondria (Figure 1f) following ariboflavinosis involved partitioning [34], whereas megamitochondria recovered after cuprizone treatment via pinching [35]. Thus, the two morphological pathways of mitochondrial division were never overlapped [33].

The ultrastructural correlate to mitochondrial fusion is less complex since it only requires a lateral alignment of the mitochondrial pair and subsequent merging of the outer and inner mitochondrial membrane, creating fewer and larger organelles [36]. Despite the fact that cardiomyocytic mitochondria are entrapped between myofibrils, they still display the potential to fuse. Apart from complete fusion, a more transient form of fusion has been observed. Liu and colleagues performed live-cell microscopic imaging of cardiac progenitor cells whilst tracking photo-activated fluorescent mitochondria [37]. The results depicted mitochondria connecting at so-called “kissing junctions”, allowing the exchange of soluble intermembrane space and matrix proteins, and re-separating, all within a period of less than four seconds. The exact reasons for these short fusions remain unclear. However, it is thought that the short fusion serves the communication between mitochondria throughout the entire mitochondrial network. In this context, Huang et al. demonstrated that the local signal of photo-activated green fluorescent protein (PAGFP) was distributed over the entire mitochondrial population within approximately 10 h [36]. Electron microscopy revealed the structural basis of the following two elemental ways of intermitochondrial communication: For one, the previously described “kissing“ in the form of extensive, intimate connections between adjacent mitochondria. For another, elongated nanotubular protrusions that link non-adjacent mitochondria to each other. These mechanisms create a dynamically continuous network to share signals for the otherwise static mitochondria in cardiomyocytes [36].

## 3. Fission, Fusion, and Mitophagy

Mitochondrial dynamics such as fission, fusion, and mitophagy are important to guarantee a homogeneous mitochondrial population, eliminate damaged and malfunctioning mitochondria, and ensure correct cell cycle activity [3]. These processes are orchestrated by a variety of functional proteins, which are encoded in the nucleus [5]. This section outlines the main functional factors and gives a brief summary of the underlying molecular mechanisms of these processes.

**Figure 2:**
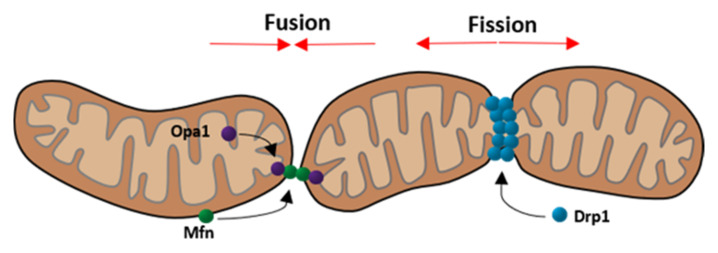
Schematic depiction of mitochondrial fusion and fission focusing on the three main components Opa1, Mfn (fusion), and Drp1 (fission). (Modified according to van der Bliek et al. [38]).

Mitochondrial fusion represents the process during which two mitochondria connect their outer and inner membranes to exchange their content (Figure 2). Two different types of fusion processes are known, namely, complete fusion and transient fusion.

During complete fusion, soluble and membrane components are exchanged between the two mitochondria. This process occurs mostly in an end-to-end connection of the involved mitochondria. While fusion takes place, the mitochondria move more slowly. After completion of the fusion process, they are elongated and move more slowly than smaller, spherical ones. In contrast, transient fusions are performed to minimize the time of reduced motility state and to maintain their morphology. During transient fusion, mitochondria are able to exchange content such as proteins or mRNA, which are necessary for mitochondrial function. Contrary to complete fusion, no exchange of membrane components takes place during transient fusion [37].

An important prerequisite for the exchange of contents between the two mitochondria is the fusion of the outer and inner mitochondrial membranes. This process is mediated by proteins as follows: The outer membrane fusion is mediated via mitofusin 1 and 2, which are part of the mitofusin family of large GTPases. In order to fuse the outer membrane, mitofusin must be present in both mitochondria [6]. The inner membrane fusion is mediated by the dynamin related GTPase optic atrophy 1 (Opa1) [7]. Opa1 exists in the following two different forms: long Opa1 and short Opa1 [39]. The long-form is located on the inner mitochondrial membrane and thereby participates in the fusion process of the inner membrane, whereas the short form is dissociated in the intermembranous space and displays the inactive form of Opa1 [28]. Mitochondrial fusion is dependent on ATP. In addition, the inner membrane fusion also depends on oxidative phosphorylation activity [7]. Mitochondrial fusion is important to ensure a homogeneous mitochondrial population and helps to eliminate the effects of mtDNA mutations [3].

The fusion process is affected by different pathologies. In this context, stress factors on the mitochondria, such as a decrease in mitochondrial membrane potential, activate the metalloendopeptidase Oma1 protein, which increases long Opa1 conversion to short Opa1. This inhibits the fusion process [5].

**Figure 3:**
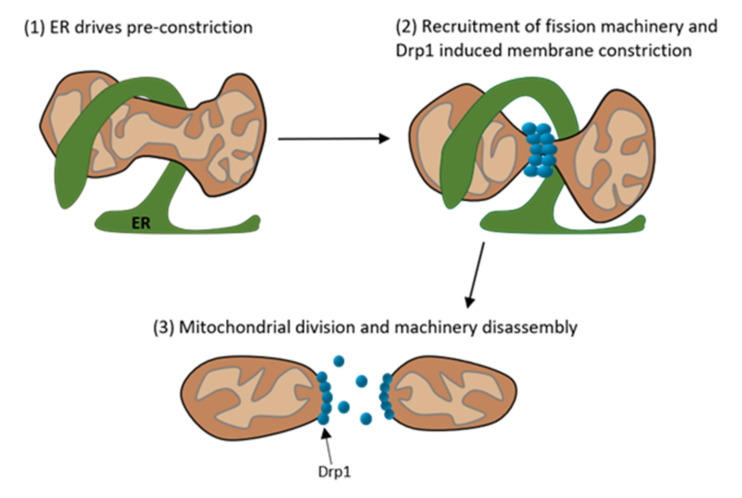
Schematic depiction of the multistep process of mitochondrial division. (**1**) The Endoplasmatic Reticulum (ER) is present at the mitochondrial division site and preconstricts the mitochondrion. (**2**) At the preconstricted mitochondria site forms the division complex with its main component Drp1, which further constricts the mitochondrion. (**3**) After the constriction the mitochondrion divides into two mitochondria and the division machinery disassembles. (Modified according to Tilokani et al. [40]).

Under physiological conditions, fusion is strictly balanced with fission. Apart from Dynamin-2 (Dnm2), fission relies mostly on the dynamin related protein 1 (Drp1) as shown in Figure 2 [5,40]. Drp1 represents the key protein for modulating mitochondrial dynamics and is even targeted by many viruses to disturb the dynamics [28]. This protein is mainly located in the cytoplasm but can be recruited to mitochondria under stress conditions [41]. The translocation is dependent on post-translational modifications, such as phosphorylation at specific sites. Two important phosphorylation sites are ser616 and ser637. While phosphorylation at ser637 keeps Drp1 in the cytoplasm, phosphorylation at ser616 promotes the translocation to mitochondria [42,43]. Drp1 not only targets mitochondria but also peroxisomes and the endoplasmic reticulum [5]. Considering this interaction, it is not surprising that fission commonly takes place at mitochondria-ER contact sites [44]. Thereby, ER tubules wrap around mitochondria and physically contract them [45]. When the diameter of the mitochondria is at an optimal size, Drp1 molecules can polymerize and form helices around the mitochondria, contracting it whereby the fission is completed [5]. This process is illustrated in Figure 3.

A study by Wakabayashi et al. emphasized the importance of mitochondrial fission by demonstrating that knockout mice with a *Drp1*−/− phenotype died intrauterine. Mitochondrial fission is especially essential for brain development. Furthermore, *Drp1* knockout in adult mice leads to elongated mitochondria, downregulation of Mfn, and cleavage of long Opa1, turning it into its inactive short form [46]. This illustrates that the inhibition of fission impacts development but does not necessarily lead to cell death. However, the inhibition of fusion leads to mitochondrial fragmentation and apoptosis [47,48]. Furthermore, studies show that the balance of all these dynamic processes is more important for cell survival than the processes themselves [3].

Another relevant process in the dynamics of mitochondria represents mitophagy, which is responsible for removing old or damaged mitochondria. There are three steps in mitophagy, namely, inhibition of the fusion process through inner membrane depolarization and following Oma1 activation (1), mitochondrial fission to segregate mitochondria and generate pieces of an appropriate size for engulfment by autophagosomes (2), and degradation of outer membrane proteins to prevent transport and fusion (3). Finally, these highly modified mitochondria can then be engulfed by autophagosomes [3].

These processes are spatially coordinated at ER membrane contact sites. Both fission and fusion proteins colocalize at ER contact sites to form hotspots for membrane dynamics. Since these hotspots can undergo fusion as well as fission, they can quickly respond to metabolic cues [30]. These mitochondria-ER contact sites (MERCS) are of additional interest since they are involved in several cellular homeostatic functions, such as lipid metabolism, calcium homeostasis, unfolded protein response, and ER stress [49]. MERCS, their associated proteins, and especially their functions, are not entirely understood.

## 4. Mitochondria in the Cardiovascular System

**Figure 4:**
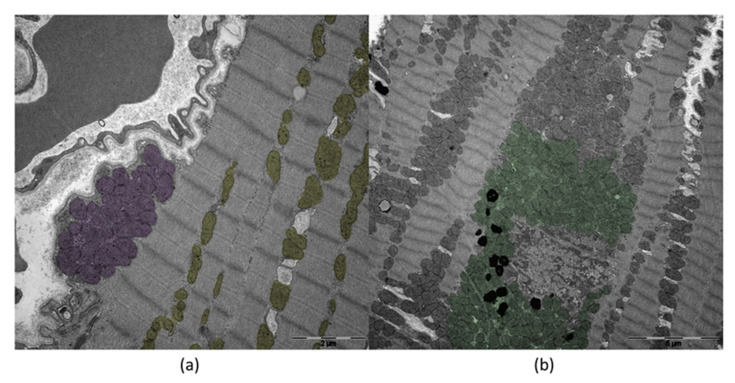
Transmission electron microscopy of human cardiomyocytes. (**a**) Subsarcolemmal Mitochondria (SSM; purple) and Intermyofibrillar Mitochondria (IFM; yellow). (**b**) Perinuclear Mitochondria (PNM; green). Institute of Pathology, Regensburg.

The mitochondrial ultrastructure in cardiomyocytes has been extensively investigated in the last decade. Physiologically, mitochondria take up about one-third of the cytoplasm of cardiomyocytes, with an average number of 6000 mitochondria per cell and an individual half-life of 5.6 to 6.2 days [50,51,52]. The morphology of mitochondria in cardiomyocytes is widely heterogeneous, which results from their compression by myofibrils. The size of mitochondria can vary within a wide range and indicates the physiological condition of the cell [53]. In the heart, they can be separated into three different subtypes depending on their location, ultrastructure, and function (Figure 4). Intermyofibrillar mitochondria (IFM) are more elongated than the other subtypes and provide energy for myocardial contractions. Energy for the ion channel function is provided by subsarcolemmal mitochondria (SSM), which are about the same size as intermyofibrillar mitochondria but more spheric [54]. The smallest group is the perinuclear mitochondria (PNM), which provide energy for gene transcription. These separate groups of mitochondria may respond differently to damage and hyperfunction of the cell [53]. For example, changes in the respiration intensity were observed for SSM but not for IFM following volume overload of the heart [55,56]. Concerning mitochondrial fission processes, SSM and IFM differ from one another as well. While mitochondria from the SSM population only divide via partitioning, interfibrillar mitochondria mainly divide through pinching and, to a small extent, also through partitioning [33].

Malfunctions of these mitochondria are linked to different pathologies in the heart. In this context, mutations in mtDNA could be linked to atherosclerosis, hypertension, diabetes mellitus, and various cardiovascular diseases [57]. Another research focus is on changed mitochondrial dynamics in acute myocardial ischemia and reperfusion injury. Ultrastructural analysis of human cardiomyocytes with heart failure and preserved or reduced ejection fraction (HFpEF/HFrEF) showed that the mitochondrial area decreased in HFpEF and even more in HFrEF compared to control hearts. Furthermore, fragmentation and cristae destruction were described in HFpEF, including vacuolar degeneration and swelling. Lysosomes were abundant in areas of mitochondrial fragmentation and could be observed during fusion with neighboring mitochondria, indicating advanced mitophagy. These findings were more pronounced in HFrEF than in HFpEF and could be seen in the IFM and PNM populations, but not in the SSM population. This was concordant with the increased expression of Drp1 and B-Cell lymphoma 2/adenovirus E1B 19 kDa protein-interacting protein 3 (BNIP3; Mitochondrial death and mitophagy marker) and the decreased expression for Peroxisome proliferator-activated receptor gamma coactivator 1-alpha (PGC-1α; Marker for mitochondrial biogenesis) [58]. This suggests a positive correlation between mitochondrial damage and the severity of heart failure. Furthermore, markers for mitochondrial death, mitophagy, and mitochondrial de novo biosynthesis exist that can possibly be correlated to morphological findings. A possible explanation for this selective damage to the PNM and IFM populations is the increased tolerance of the SSM population towards oxidative stress [59]. It is notable, that hypoxia-inducible factor 1-alpha (HIF-1α) is activated in ischemic heart diseases and increases mitochondrial fission [60,61].

In a transgenic murine model, cardiac hypertrophy was simulated by overexpression of myotrophin (a gene responsible for muscle growth) and compared with age-matched wild-type (WT) mice [62]. The ultrastructural study of murine cardiomyocytes showed several changes in mitochondrial morphology. While no structural abnormalities could be seen during the initiation phase (4 weeks after initiation), loss of mitochondrial granules and swelling were identifiable in the progression phase (12 weeks after initiation). The depletion of mitochondrial granules has both been connected to settings of hypoxia and states of high oxygen stress [63]. Other studies have indicated that mitochondrial swelling and translucent spaces between cristae occur when ATP content is lost [61]. In the transition phase from hypertrophy to heart failure (a minimum of 34 weeks after initiation), loss of matrix granules, and swelling of the organelles increased significantly. Additionally, more and more mitochondria, including concentric cristae, were visible. The ratio of swollen to normal mitochondria rose with the increasing severity of heart failure. Notably, aged WT mice also exhibited a few swollen mitochondria, suggesting higher energy demand to be a natural consequence of the aging process [62].

An electron-microscopy study of rat myocardium 2 weeks and 6 months after experimental myocardial infarction by ligation of the left coronary artery with chronic heart failure (CHF) demonstrated a substantial decrease in the IFM population and the density of cristae [64]. Swollen mitochondria were observed both in the border zone of the infarct and the intact zone, indicating the development of ischemia as a whole. Mitochondria formed prismated structures and had a 30% decrease in the density of cristae compared with physiological probes. In CHF, occasional large megamitochondria—which can measure up to 14 μm [65]—could be found, which was discussed as a compensatory mechanism. It has been suggested that such large organelles are formed in response to short-term non-lethal hypoxia [66,67,68]. It is noteworthy that 6 months after the myocardial infarction, the mitochondrial ultrastructure had resumed its physiological morphology, except in rats with CHF. These still exhibited vastly swollen and degenerating mitochondria with reduced density of the arrangement of the inner mitochondrial membranes, which could be interpreted as a hint of a decrease in energy metabolism in cells of the border zone of the infarct. However, along with the decrease in metabolism, a reduced production of reactive oxygen species is described, which is discussed as beneficial [69].

Since cardiovascular diseases are the major cause of death in developed countries with a focus on ischemic heart disease [70], new therapies need to be developed to reduce myocardial infarct size, preserve left ventricular function, and prevent the transition to terminal heart failure [71]. Proteins, which are involved in the dynamics of mitochondria, have become more and more the focus as promising target molecules. In this context, it could already be demonstrated that an acute inhibition of fission or elevation of mitophagy has a cardioprotective effect after an acute myocardial infarct [72]. The overall strategy to decrease cardiac damage after an acute myocardial infarct is to prevent mitochondrial dysfunction during acute myocardial ischemic reperfusion injury. This can be achieved by reducing the mitochondrial size by targeting fission and fusion proteins to reduce fission. In electron microscopic studies, it could be shown that fission is increased in myocardial ischemia/reperfusion injury (IRI), myocarditis, stroke, doxorubicein cardiotoxicity, sepsis-related cardiomyopathy, post-AMI cardiomyopathy, and diabetic cardiomyopathy. These morphological changes are caused by oxidative stress, p38 mitogen-activated kinase (p38 MAPK), Cyclin-dependent kinase 1 (Cdk1), Protein kinase C delta type (PKC-δ), calcium overload, calcineurin, SUMOylation of Drp1, succinate, lower myocardial levels of dual-specificity protein phosphatase1 (DUSP1), and the upregulation of nuclear receptor subfamily 4 group A member 1 (NR4A1) [73].

First, the experimentally used quinazolinone derivative mitochondrial division inhibitor 1 (midivi-1), which is a Drp1 GTPase inhibitor, was shown to reduce cell death in isolated murine cardiomyocytes and reduce myocardial infarct size in the murine heart after myocardial IRI [74]. Even though the inhibition of Drp1 was shown to be cardioprotective, midivi-1 was also shown to have side effects resulting from the inhibition of the respiratory chain complex I [75,76]. More precise inhibitors were developed, for example, Driptor 1, which inhibits the GTPase activity of Drp1, genetic inhibition of Drp1 with the microRNA miR499, which prevents Drp1 translocation through the suppression of Drp1 dephosphorylation, and adenovirus expressing Drp1 dominant negative K38A, which was used to induce loss-of-function in Drp1 [4,75,77]. Not only can the blocking of Drp1 directly inhibit mitochondrial fission but also agents such as Dynasore, which is a dynamin inhibitor, and the synthetic peptide P110, which inhibits the Drp1 and hFis interaction [78].

The application of a fission inhibitor should not be chronic. It was shown that the best time for the use of these agencies is at the onset of reperfusion [12,78]. Since the timing of administration is so important, the delivery to the ischemic part of the heart must be fast and precise. A study by Ishikita showed how a newly developed nanoparticle delivery method is successful in delivering midivi-1 to the ischemic part of the heart [11].

Enhancing mitophagy during acute myocardial ischemia and reperfusion (I/R) injury is also cardioprotective [71]. Not only can myocardial infarct size be influenced by these mechanisms, but also coronary microvascular injury. This was shown with the cardioprotective factor melatonin, the sodium glucose cotransporter 2 inhibitor empagliflozin, and the Bax inhibitor, BI1, which interacts with the Spleen tyrosine-kinase (Syk; non-receptor tyrosine-kinase) pathway [79,80,81]. This shows that inhibiting mitochondrial fission and increasing mitophagy has a cytoprotective effect on both cardiomyocytes and the coronary microvasculature.

**Figure 5:**
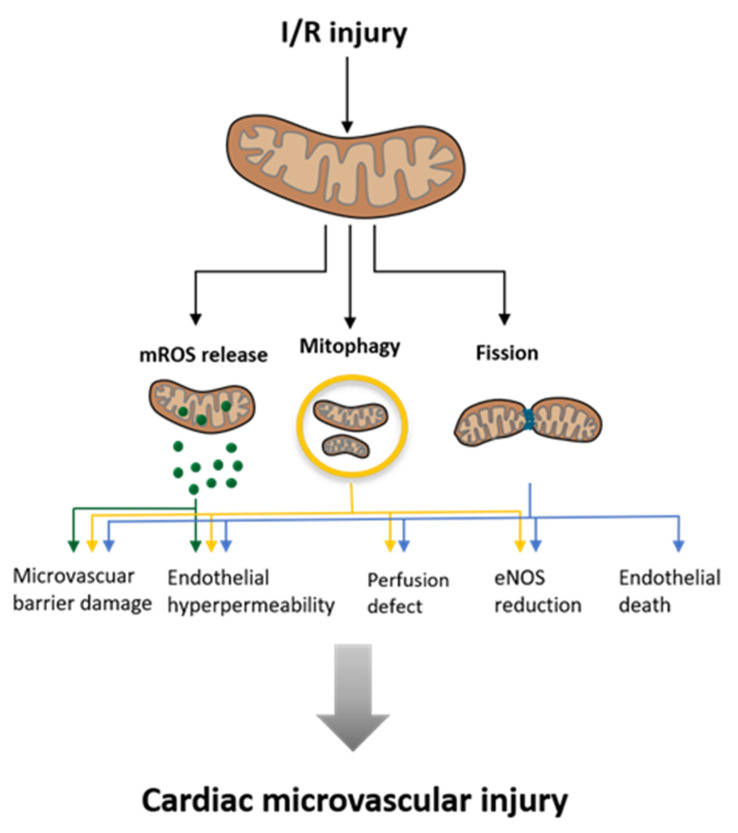
Influence of Ischemia and Reperfusion (I/R) injury on mitochondria and the consequences for the surrounding cardiac microvascular tissues. (Modified according to Wang et al. [88]).

Mitochondrial fission has been widely explored in the context of cardiomyocyte I/R injury, but the functional contribution to endothelial damage and regulatory mechanisms is still not fully understood [82]. Nevertheless, different mechanisms involved in fission processes are linked to I/R injury and heart failure, schematically represented in Figure 5. Ischemia in cardiomyocytes leads to a lack of oxygen. To supply energy, mitochondria start glycolysis, which leads to an accumulation of pyruvate. Pyruvate is converted to lactate, a process which is linked to the conversion of Nicotinamid adenine dinucleotide hydride (NADH) to Nicotinamid adenine dinucleotide (NAD+) and a proton (H+), consequently leading to intracellular acidification. Following acidification, mitochondrial transition pore opening is prevented and a secondary Na+ influx, as well as a Ca2+ accumulation takes place. When reperfusion takes place, the pH in the mitochondria normalizes and the pores open, which leads to a ROS burst and an uncontrolled influx of molecules smaller than 1.5 kDa. This leads to a collapse of the mitochondrial membrane potential and subsequent swelling and cell death [83]. ROS production seems to have a relevant impact on cell injury procedures in I/R injury. Mitochondrial fission promotes mROS production and vice versa [82]. It could also be shown that under I/R injury, Drp1 is primarily phosphorylated at Ser616 and dephosphorylated at Ser637 [84], which leads to a Drp1 accumulation around the mitochondrial outer membrane, leading to fission [82]. The link between ischemia and ROS production became even more clear by the finding that the acetylation of SDHA in ischemia leads to the oxidation of succinate, which contributes to ROS production in reperfusion [85].

While this article mainly focuses on cardiomyocytic mitochondria, it is mentionable that these organelles are crucial for the functionality of other cardiovascular cell types, such as vascular endothelial cells (ECs) or vascular smooth muscle cells (VSMCs). Mitochondrial malfunction is linked to certain pathologies in these cell types as well. For example, mitochondrial dysfunction in VSMCs is thought to be involved in the pathomechanism of vascular calcification [86]. In ECs, mitochondria are vital not only for energy supply but also for responsive signaling following environmental cues [87]. In I/R-injury cellular damage occurs to the ECS before it occurs to cardiomyocytes. As aforementioned, this damage is mediated by mitochondrial fission and leads to microvascular injury as illustrated in Figure 5.

The functional interaction between ischemia, defective mitochondria, and ROS production has been strongly investigated in cardiomyocytes, and promising treatments targeting these aspects have been proposed. Especially the inhibition of mitochondrial fission is auspicious to reduce cell mortality in I/R injury. With a view to increasing antibiotic resistance, the possible use of similar agents in infectious diseases should be explored.

## 5. Mitochondrial Dynamics in the Musculoskeletal System

Despite the growing importance of mitochondrial fission and fusion for cell physiology and pathophysiology in the cardiovascular system, it has only been scarcely investigated in trauma surgery and orthopedics. However, a few studies have already elucidated the alteration of mitochondrial dynamics in certain orthopedic diseases and suggest possible therapeutic targets. So far, mostly expression analyses of fission and fusion markers have been performed, as well as fluorescent live-cell imaging in animal models. Ultrastructural analyses via transmission electron microscopy (TEM) of bone cells are rare. Furthermore, it lacks an overall classification and description of osteoblastic and osteoclastic mitochondria. Nevertheless, there is some evidence for the functional relevance of mitochondrial dynamics in several musculoskeletal diseases, such as osteoporosis, diabetes, and osteomyelitis.

Osteoporosis, a common disease of the elderly, is characterized by an increase in bone resorption by osteoclasts and a decrease in bone formation by osteoblasts [89], resulting in net bone loss and increased bone fragility [90]. Apart from estrogen deprivation, aging and its concomitant accumulation of oxidative stress have been identified as key protagonists in the pathogenesis of osteoporosis [91]. Oxidative stress-induced osteoblast dysfunction is thought to be a major factor in bone loss [92]. Furthermore, it contributes to the inhibition of osteoblast differentiation and proliferation and induces cell death [93,94,95]. Since—apart from the enzyme Nicotinamid adenine dinucleotide phosphate oxidase (NADPH oxidase)—mitochondria represent a major source of oxidative stress due to the generation of ROS these organelles are actively involved in the underlying pathomechanisms, given by the mitochondrial fragmentation [96,97,98]. The pharmaceutical manipulation of the dynamics of mitochondria promises to have an impact on the outcome of osteoporosis. In an in vitro study on osteoporosis, H_2_O_2_—as a representative molecule for oxidative stress—induced osteoblastic dysfunction through upregulation of mitochondrial fission [99]. This could be seen in an increase in the phosphorylation of Drp1 at Ser616. Experimental blockade of Drp1 rescued a number of osteoblasts, which became evident from increased cell viability, improved cellular alkaline phosphatase (ALP) activity, restored mineralization, and mitochondrial function. Thus, intervention in mitochondrial dynamics indicates a potential new therapeutic strategy for the treatment of osteoporosis, which needs further experimental and animal studies.

Another metabolic disease, diabetes, also comes along with increased bone fragility and fracture risk [100]. This is of particular interest because the bone mineral density (BMD) is elevated instead of decreased. Pahwa et al. proposed that the reduced cell motility of osteoblasts following hyperglycemic conditions leads to an abnormal distribution of bone extracellular matrix (BEM) [101]. In this context, it has been shown that silencing of Drp1 and the subsequent inhibition of mitochondrial fission, leads to a reduction in the migratory potential of cancer cells [102]. Thiel et al. demonstrated that the migration potential is also vital for osteoblastic functionality since it is required for the synthesis of an optimal bone structure [103]. In an in vitro study, Pahwa et al. elucidated that constant hyperglycemic conditions similar to those of diabetic patients were followed by a decrease in the expression of Drp1 and mitochondrial biogenesis markers in osteoblasts [101]. The authors concluded that impaired mitochondrial dynamics may be the cause of decreased osteoblastic cell migration and chemotaxis and thus be a relevant factor in the development of fragile bones in diabetes (101).

A challenging disease within the musculoskeletal system represents osteomyelitis. Despite modern therapies such as antibiotics and highly developed surgical techniques, bacterial osteomyelitis still remains difficult to treat. Furthermore, the chronification rates remain significantly high [104]. After numerous surgical excisions of avital and infectious tissue, amputation often remains the last option for patients suffering from osteomyelitis [105]. Two forms of chronification have been observed. For one, the forming of so-called biofilms on the foreign matter or necrotic tissue, and for another, the internalization of bacteria by osteoblasts [106] and the subsequent evasion of immune cells [107]. In this context, *Staphylococcus aureus (staph. aureus)* has been described as the most common pathogenic germ in osteomyelitis [108,109]. Although less is known about the impact on the host cell’s metabolism by intracellular bacterial pathogens (IBPs) than viral pathogens, it is evident that IBPs persist in host cells via a bipartite metabolism [110,111], in which the high-energy C3-metabolites are extracted from the cytosol and mainly compounds that are not supplied by the host cell are synthesized de novo by the IBPs [112]. The subsequent depletion of ATP in the host cell would theoretically lead to increased activation of AMPK and thereby induce mitochondrial fission [82].

In addition, there is evidence that bacterial infections are closely related to microenvironmental tissue hypoxia [113]. Since hypoxia decreases the local energy supply it is clear that the AMPK is activated in infections without intracellular bacteria [114,115]. The main mammalian response to hypoxia is the activation of hypoxia inducible factor 1α (HIF-1α). In this context, it could be demonstrated that HIF-1α increases the fission rate by a Cyclin B1/CDK1-dependent phosphorylation of Drp1 at Ser616 [60]. Furthermore, in an animal study on osteomyelitis caused by *staph. aureus* a downregulation of fusion-related genes such as *OPA1* could be seen together with an up-regulation of fusion-inhibiting *OMA1*. Fission-related genes, on the other hand, displayed a slight upregulation. This shift in the balance between mitochondrial fusion and fission indicates mitochondrial dysfunction [116]. Since mitochondrial fragmentation is closely related to autophagy and apoptosis [117], mitochondria can be considered as a switching point between withstanding the bacterial infection and cell death, which would ultimately lead to an outbreak of the chronic disease and deterioration of symptoms such as bone fragility due to decreased bone matrix synthesis [118].

How and to what extent the manipulation of mitochondrial dynamics could potentially alter the outcome of chronic osteomyelitis should be analyzed more in detail in future studies.

## 6. Mitochondrial Dynamics as a Potential Link between Ischemia and Osteomyelitis

So far, little is known about the dynamics of mitochondria in osteomyelitis and whether they could be altered to improve the clinical outcome of this important disease. In contrast, myocardial ischemia has been readily investigated with a large focus on mitochondrial fission and fusion, and the question arises whether or not knowledge from the therapeutic modification of mitochondria in the cardiovascular system is transferable to the musculoskeletal system.

From a pathophysiological point of view, the first connection between these two fields is given by the depletion of ATP in the afflicted cells. Hypoxia in the heart gradually diminishes the ATP resources of the cell since the more efficient aerobic metabolic pathway is inhibited due to the lack of oxygen [119,120]. In bacterial osteomyelitis, an increasingly discussed pathomechanism is the internalization of bacteria by osteoblasts and osteocytes, and thus the evasion of immune cells [121,122,123,124]. So far, the evidence concerning intracellular bacterial pathogens (IBPs) in osteomyelitis is restricted to case reports; therefore, it is of importance to further determine the clinical relevance of such findings [106,125]. However, IBPs draw energy-rich C3-metabolites such as pyruvate or glycerol from the cytoplasm [111] and subsequently lead to a breakdown in the cellular ATP supply. An increase in the AMP:ATP ratio allosterically activates the AMP-activated kinase (AMPK). Since the activation of the AMPK provides intracellular metabolites that improve pathogen replication, it is assumed that IBPs might directly stimulate the AMPK [126]. As mentioned above, osteomyelitis displays local tissue hypoxia, as is commonly found in inflammation associated with bacterial infection [115]. This suggests that AMPK should also be activated in bacterial infections lacking the presence of IBPs [114]. Furthermore, as local tissue hypoxia impedances cell proliferation of most cell types [127], it may be a disadvantage that infiltrating inflammatory cells and inflamed resident cells contribute to diminished bone stability and the chronification of the disease.

**Figure 6:**
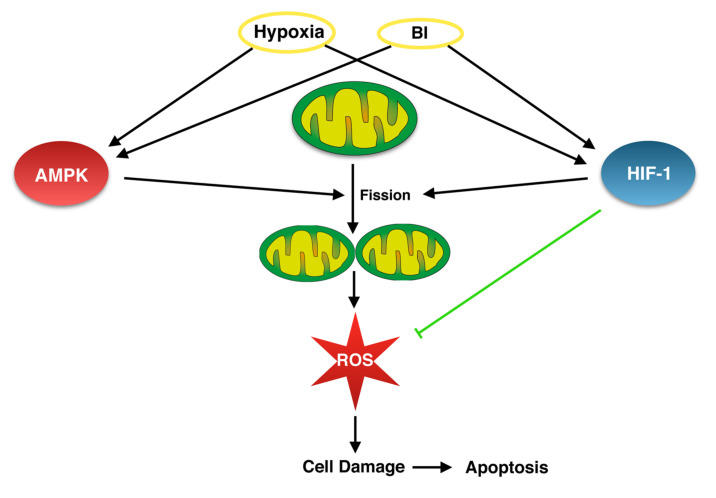
Hypothetic link between bacterial infections (BI) and hypoxia via AMP-activated kinase (AMPK) and Hypoxia-inducible factor-1-alpha (HIF-1α) pathways, which should be proven experimentally to improve our understanding of the cellular mechanisms in osteomyelitis.

An in vitro study with an osteosarcoma cell line demonstrated that an up-regulated AMPK-pathway triggers mitochondrial fragmentation [128]. This was either achieved indirectly through the depletion of ATP via the inhibition of complexes I and III of the electron transport chain (ETC) or by the direct activation of AMPK. Hypothetically, one can find an upregulated AMPK in ischemic cardiomyocytes as well as in inflamed bone cells associated with bacterial infection and, subsequently, an increase in mitochondrial fission, which may be a key factor in the cell damage (Figure 6).

Another connection is the activation of hypoxia-inducible factor 1-alpha (HIF-1α). Under normoxic conditions, the alpha subunit (HIF-1α) is constantly degraded, whereas under hypoxia, the degradation is inhibited and HIF-1α accumulates to merge with the beta subunit [129]. HIF-1α then migrates to the nucleus and promotes the expression of several genes, including genes involved in cell proliferation and survival as well as glucose and iron metabolism [130]. Interestingly, bacterial infections are generally associated with the activation of HIF-1α [113,131]. Despite not yet being investigated in osteomyelitis, one can assume that HIF-1α would also be activated due to the consistency of this finding in other infections with human pathogens. The genetic deletion of *Hif1a* in bacterial peritonitis caused by *Staph. aureus* in a murine model showed promising results, with a significantly improved survival rate of *Hif1a* knockout mice [132].

However, the inhibition of HIF-1α in osteomyelitis might bring along adverse effects. In a model of endodontic infection resembling osteomyelitis, the inhibition of HIF-1α was followed by a reduction in reactive bone formation [133]. Furthermore, HIF-1α itself contributes to the reduction of mitochondrial-derived oxidative stress [134]. Thus, something further downstream appears to be more promising for the following treatment of osteomyelitis: mitochondrial fission. As is the case for several other metabolic factors, activated HIF-1α effects mitochondrial dynamics [60]. This is achieved by promoting fission via a CDK1/Cyclin-B-dependent pathway [97].

It is thought that an increase in mitochondrial fission leads to a rise in the production of cellular reactive oxygen species (ROS) and vice versa [97]. ROS is responsible for cell damage and thereby triggers cell death pathways such as apoptosis [135]. In the cardiovascular system, it has already been demonstrated that inhibiting mitochondrial fission can protect the heart against I/R-injury, potentially by preventing the intensification of ROS production [136]. Although the exact mechanisms by which IBPs induce cell death in osteomyelitis are not yet completely understood, it is acknowledged that their most common pathogen—*Staph. aureus*—induces host cell death mainly by apoptosis [137]. Being strongly linked to apoptosis, mitochondria offer a potential target to modulate the physiology of the cell. In this context, limiting mitochondrial fission could protect infected osteoblasts from ROS overproduction and apoptosis and, in due course, grant antibiotics the possibility to destroy intracellular bacteria before the death of the host cell and the subsequent release of the pathogens.

In conclusion, the link between hypoxia and osteomyelitis opens innovative perspectives for a better understanding of the pathomechanisms of osteomyelitis. In this context, one would expect ultrastructural correlates such as increased mitochondrial mass involving hydropic and even prismated mitochondria and several correlates of mitochondrial damage in infectious bone samples, which are topics of ongoing research in our group. Furthermore, the modulatory strategies coming from the cardiovascular system should be investigated more in detail with a view to osteomyelitis to further optimize the therapeutic strategies in this complex disease. In this context, the experimental inhibition of mitochondrial fission displayed cell-saving effects in cardiac ischemia. If the link between hypoxia and osteomyelitis is indeed verified, the same inhibition of fission in osteomyelitis may unleash osteoblasts’ and osteocytes’ regenerative potential, which potentially could support healing mechanisms in osteomyelitis. Thus, the comparison and translation of mitochondrial dynamics from the cardiovascular system to bone infection opens innovative perspectives in both a better understanding of pathomechanisms and potential new treatment options.

## Data Availability

Not applicable.
